# Multicoverage Study of Femtosecond Laser-Induced Desorption of CO from Pd(111)

**DOI:** 10.1021/acs.jpclett.4c00026

**Published:** 2024-02-28

**Authors:** Alberto S. Muzas, Alfredo Serrano Jiménez, Yaolong Zhang, Bin Jiang, J. Iñaki Juaristi, Maite Alducin

**Affiliations:** † Departamento de Polímeros y Materiales Avanzados: Física, Química y Tecnología, Facultad de Químicas (UPV/EHU), Apartado 1072, 20018 Donostia-San Sebastián, Spain; ‡ Centro de Física de Materiales CFM/MPC (CSIC−UPV/EHU), Paseo Manuel de Lardizabal 5, 20018 Donostia-San Sebastián, Spain; § Hefei National Laboratory for Physical Science at the Microscale, Key Laboratory of Surface and Interface Chemistry and Energy Catalysis of Anhui Higher Education Institutes, Department of Chemical Physics, University of Science and Technology of China, Hefei, Anhui 230026, People’s Republic of China; ∥ Donostia International Physics Center (DIPC), Paseo Manuel de Lardizabal 4, 20018 Donostia-San Sebastián, Spain

## Abstract

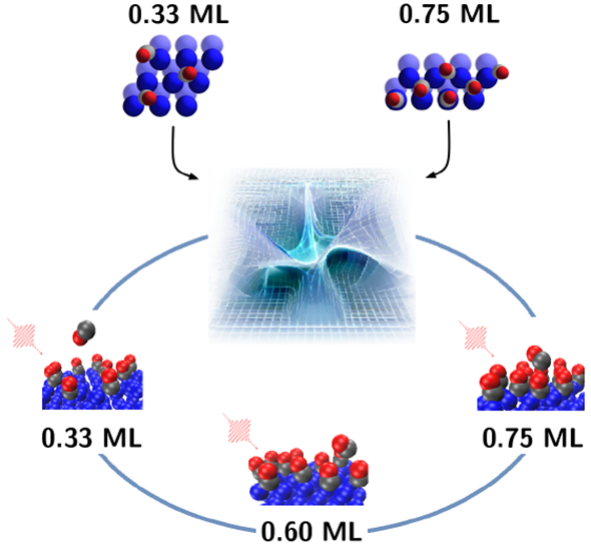

We study the strong coverage dependence of the femtosecond laser-induced desorption of CO from Pd(111) using molecular dynamics simulations that consistently include the effect of the laser-induced hot electrons on both the adsorbates and surface atoms. Adiabatic forces are obtained from a multicoverage neural network potential energy surface that we construct using data from density functional theory calculations for 0.33 and 0.75 monolayer (ML). Our molecular dynamics simulations performed for these two trained coverages and an additional intermediate coverage of 0.60 ML reproduce well the peculiarities of the experimental findings. The performed simulations also permit us to disentangle the relative role played by the excited electrons and phonons on the desorption process and discover interesting properties of the reaction dynamics as the relevance that the precursor physisorption well acquires during the dynamics as coverage increases.

The study of adsorbates on top of metallic surfaces under the action of high-fluence femtosecond (fs) laser pulses poses an excellent field to probe our understanding of how hot electrons and phonons influence fundamental gas–surface reactions, including desorption. When the fluence of the incident laser pulse surpasses a certain threshold value [e.g., 10 J/m^2^ for O_2_ photodesorption on Pt(111)], desorption induced by multiple electronic transitions (DIMET) is the predominant reaction mechanism.^1^ Under this regime, photons reaching the decorated metallic target mostly interact with electrons in the substrate, creating electron–hole pairs that can populate excited adsorbate–surface states. These excited complexes quickly decay by the coupling with resonant metallic excited electronic states with typical quenching times of 1–100 fs,^2^ leaving often kinetically excited adsorbates after this relaxation. Thanks to the high density of electronic excitations, multiple adsorbate excitation–deexcitation cycles can occur before complete adsorbate relaxation. This yields an effective “ladder climbing” mechanism that eventually pushes for adsorbates to overcome reaction barriers.^3^ At the same time, these hot electrons experience electron–electron and electron–phonon scattering processes, which produce quick electronic relaxation until reaching a hot Fermi–Dirac distribution and gradual surface lattice heating. Hot phonons created in this way can subsequently compete or collaborate with the pure DIMET mechanism to promote desorption.^4−7^

Theoretically, the aforementioned processes can be efficiently modeled on metals using stochastic molecular dynamics with electronic friction (MDEF) approaches.^3,8−10^ These type of models rely on the quick formation of a Fermi–Dirac distribution of excited electrons that thermally interact with adsorbates through electronic-friction-dependent drag and random forces, with the rest of interactions being treated under an adiabatic (Born–Oppenheimer) formulation. The heating of electrons by the laser pulse is assumed to proceed as prescribed by the two-temperature model (TTM).^11^ The suitability of these approaches (MDEF + TTM) for DIMET modeling has been supported by their success in reproducing the main features observed in fs laser pulse experiments. Some of these DIMET “hallmarks” are the superlinear power law dependence of desorption/reaction yields with laser fluence (i.e., *Y* ∝ *F*^*n*^, with *n* > 1), the quasilinear increase of kinetic energy of desorbates with laser fluence, and the presence of isotope effects in desorption/reaction yields.^12^ Many of these typical features have been studied in systems such as CO on Cu(100),^13−15^ Ru(0001),^6^ and Pd(111),^7,16^ H_2_ (D_2_ and HD) on Ru(0001),^17−19^ and O_2_ on Ag(111).^5^ More recently, also the branching ratio of CO_2_ formation and CO desorption on a CO + O covered Ru(0001)^20−22^ surface predicted with *ab initio* MDEF simulations that account for not only the laser-induced electronic excitations but also the hot lattice, as prescribed by TTM (hereafter, (*T*_e_, *T*_l_)–AIMDEF or (*T*_e_, *T*_l_)–MDEF, depending upon whether adiabatic forces are calculated *ab initio* or through a potential energy surface), have been found to be in good agreement with experiments.^23^ Furthermore, molecular dynamics (MD) techniques combined with TTM have been used to describe related phenomena, such as photodamage, melting, and surface ablation, by short laser pulses.^24−27^

Despite the abundance of MDEF studies applied to DIMET modeling, among them, there is a lack of systematic evaluation of how adsorbate coverage affects photodesorption features. Changing the structure of surface adlayers can modify adsorbate–adsorbate and adsorbate–surface interactions that are important for the dynamics of the desorption process. For instance, it has been found experimentally that CO surface coverage has a dramatic impact on the CO single-pulse fs laser-induced photodesorption yield on Pd(111), which can vary by orders of magnitude depending upon the initial coverage.^28^ Not only may the final desorption yield vary but also the relative role that hot electrons and phonons play during the process. This has been explored in the same CO/Pd(111) study using two-pulse correlation experiments,^28^ where the width of the correlation signal is expected to provide insights on the time scale of the photoinduced reaction and, therefore, on whether the underlying adsorbate–substrate energy transfer mechanism is electron- or phonon-mediated. Their results showed noticeable changes in the time scale of the desorption process as a function of coverage, yet the fundamental energy exchange mechanism could not be unambiguously determined. The measured correlation widths, which varied in the range of 11–34 ps, were compatible with both a weak adsorbate–hot electron coupling or a strong adsorbate–phonon coupling. Puzzlingly, a subsequent analysis with an empirical friction three temperature model^4,29,30^ suggested that phonons exerted little to no influence on the final shape of the two-pulse correlation desorption yields for the whole range of studied CO coverages, despite the high lattice temperatures predicted by the model. To shed light on the matter with a MDEF approach, the main challenge to beat is the construction of a potential energy surface (PES) or force field that is able to describe with accuracy the interaction of the adsorbate (CO) and substrate (Pd) for a wide range of coverages. This problem can be solved using a transferable^31^ embedded atom neural network (EANN) potential^32^ to extract adiabatic forces during dynamics.

In this work, we have developed and used an accurate multicoverage EANN PES together with an improved implementation of the (*T*_e_, *T*_l_)–MDEF approach to study the impact of CO coverage on photodesorption of CO from Pd(111) in the DIMET laser fluence regime. Within this improved framework (see the Supporting Information(33) for a detailed description), the interaction of the impinging laser pulse with the metallic substrate is modeled by the heating of two thermal baths: one for the substrate electrons with temperature *T*_e_(*t*) and the other for substrate lattice vibrations with temperature *T*_l_(*t*). The dynamics of the heat exchange between *T*_e_(*t*) and *T*_l_(*t*) in the presence of the laser pulse that is only allowed to heat electrons is computed using a set of coupled TTM heat equations^11^ parametrized for Pd.^33^ (*T*_e_, *T*_l_)–MDEF calculations are subsequently performed with all CO and Pd atoms attached to a Langevin thermostat that depends upon electronic friction coefficients (drag and random forces) and *T*_e_(*t*) (random forces only). C and O electronic friction coefficients are obtained using the local density friction approximation (LDFA),^9,34^ while Pd friction coefficients are evaluated to be consistent with the electron–phonon coupling constant utilized in the TTM model. As shown in the Supporting Information,^33^ the resulting lattice temperature coincides rather well with the temperature obtained from TTM. The advantage of this new implementation is that it improves the consistency of the (*T*_e_, *T*_l_)–MDEF methodology, which, in previous works, relied on a Nosé–Hoover thermostat attached to surface atoms at temperature *T*_l_(*t*).^7,16,35^ In the implementation presented here, surface heating is directly driven by *T*_e_(*t*) and Pd friction coefficients. A crucial ingredient to investigate the dependence of the CO photodesorption dynamics upon the initial coverage is to count with a reliable multicoverage CO/Pd(111) PES that is able to describe accurately the adiabatic interactions irrespective of coverage. In our case, adiabatic forces are obtained from an EANN PES trained with mixed configurations extracted from prior (*T*_e_, *T*_l_)–AIMDEF calculations made for 0.75 and 0.33 monolayer (ML) CO coverages, as described in the Supporting Information.^33^ All in all, the remarkable level of accuracy reached by the final EANN PES is characterized by fitting energy root-mean-square errors (RMSEs) of less than 1 meV per moving atom and less than 60 meV/Å per atomic force component, with respect to the full (*T*_e_, *T*_l_)–AIMDEF data set composed of about 485 000 configurations of mixed coverages. As shown in the Supporting Information,^33^ also the minimum energy paths (MEPs) calculated in ref (7) for desorption of one CO from each of the covered surfaces are well-reproduced by our multicoverage EANN. As a note in passing, the MEPs and AIMDEF calculations were performed with density functional theory (DFT) and the exchange correlation functional by Dion et al.^36^ that correctly described the experimental adsorption structures, even if the experimental adsorption energy still seems overestimated by about 0.2 eV (see ref (7) for a detailed discussion). Interestingly, only for CO adsorbed on the face-centered cubic (fcc) and hexagonal close-packed (hcp) sites at 0.75 ML is the physisorption clearly identified. In all cases, however, the CO–surface interaction is dominated by the chemisorption well as also found for CO co-adsorbed with O on Ru(0001)^37^ but not for CO on Au(111), which is dominated by physisorption.^38^ The ability of this type of neural network to learn interaction energies in an additive fashion from the environment of individual atoms makes the trained PES flexible enough^31^ to describe intermediate coverages, as we will show in detail latter in this letter. At this point, let us remark that an error analysis of energies and forces for random configurations visited in 0.60 ML (*T*_e_, *T*_l_)–MDEF trajectories shows RMSE values that are roughly the same as those obtained in the PES quality check against 0.75 and 0.33 ML configurations.

To explore the DIMET photodesorption process for low, medium, and high CO coverages, we have performed (*T*_e_, *T*_l_)–MDEF calculations for three different initial coverages, namely, 0.33, 0.60, and 0.75 ML, using absorbed laser fluences ranging from 50 to 130 J/m^2^. Figure 1 shows the optimized adlayer structure and supercells used in our dynamics simulations for each coverage. For the low and high coverages, we additionally performed dynamics calculations with frozen lattice atoms (hereinafter called *T*_e_–MDEF simulations) and dynamics calculations with adsorbate electronic friction coefficients set to zero (hereinafter called *T*_l_–MDEF simulations). These additional simulations are aimed to evaluate the individual impact of hot electrons and hot lattice atoms on CO photodesorption and its possible dependence upon coverage. Initial configurations in all cases were chosen randomly from coverage-specific ensembles of 100 independent (*T*_e_, *T*_l_)–MDEF trajectories thermalized to *T*_e_ = *T*_l_ = 90 K. Each thermalized trajectory was initiated from its EANN PES optimal configuration with randomized initial velocities and integrated up to 50 ps. All configurations sampled before reaching a Boltzmann distribution (from 0 to 15 ps, approximately) were discarded. Interestingly, during 0.33 and 0.75 ML thermalizations, CO diffusion was not observed. For these two coverages, adsorption site proportions fcc:hcp:bridge:atop (sorted in descending adsorption strength order) were conserved throughout the thermalization process without regard of the supercell used, specifically, 1:0:0:0 for 0.33 ML and 1:1:0:1 for 0.75 ML (see Figure 1). In contrast, in the case of 0.60 ML, CO molecules adsorb preferentially in a combination of bridge and 3-fold hollow sites (fcc and hcp), whose proportions vary during thermalization dynamics, despite the low thermalization temperature (90 K), highlighting the mobility of CO at this intermediate coverage. It is worth remarking at this point that the thermalization dynamics already serves to confirm the quality of the EANN PES. All of the thermalization trajectories started with the *c*(5 × √3)rect-3CO structure proposed by experimentalist for this intermediate coverage,^28,39^ with 1:0:2:0 being the optimized proportion that we obtain after relaxation. Despite this, the analysis of the configurations that are sampled during those thermalization trajectories shows that our EANN PES predicts a complex (5 × 2√3)rect-12CO structure with adsorbing site proportions of 2:1:3:0 that is more stable than the initial structure. The two structures are compared in the bottom panels of Figure 1. As detailed in the Supporting Information,^33^ this prediction was further corroborated by DFT calculations, in which the new *c*(5 × 2√3)rect-12CO structure found by the EANN PES is also a local minimum more stable than *c*(5 × √3)rect-3CO. This is an additional remarkable test of the accuracy of our multicoverage EANN PES, because it was not trained with configurations coming from 0.60 ML AIMDEF dynamics. As a final remark, note that the existence of several stable structures is in line with a variety of low-energy electron diffraction patterns that were identified in this system at 100 K as the coverage varies from 0.5 to 0.6 ML.^39^

**Figure 1:**
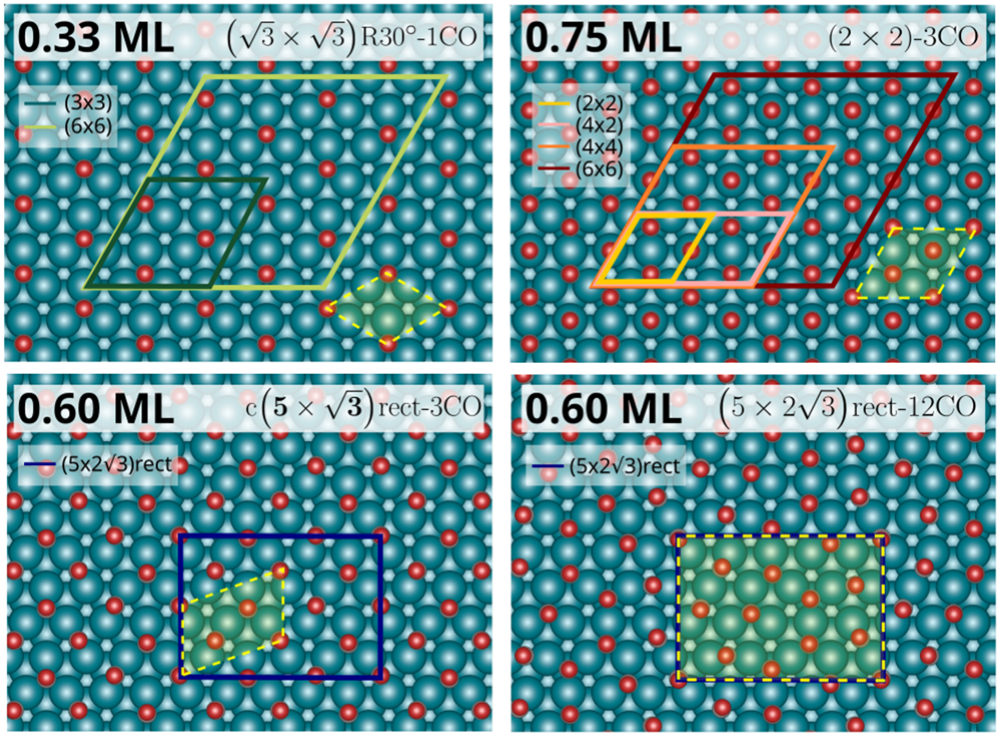
Top view of minimum energy configurations calculated with the EANN PES for 0.33 ML (top left), 0.75 ML (top right), and 0.60 ML (bottom left and right) of CO coverage. In the latter case, the bottom left structure corresponds to a local minimum higher in energy than the bottom right structure. The actual supercells used in the (*T*_e_, *T*_l_)–MDEF simulations are represented in full lines. Yellow dashed lines and shaded areas show the smallest pattern that is repeated within supercells.

The main results of our simulations regarding the dependence of the CO photodesorption probability *P*_des_ upon laser fluence *F* and coverage are summarized in Figure 2. Each data point is calculated from a set of 6750 to 60 000 CO trajectories, depending upon the statistical needs, that are integrated up to 100 ps, using the Beeman algorithm and 0.2 fs as the integration step. The left panel shows the dependence of *P*_des_ upon the absorbed laser fluence. Our (*T*_e_, *T*_l_)–MDEF results for CO coverages of 0.75, 0.60, and 0.33 ML are plotted in reddish, bluish, and greenish full circles, respectively. Following the same color code, we show previous experimental results^28^ with full triangles but in this case for 0.75, 0.64, and 0.24 ML of CO. For each data set, the log–linear fit used to extract the exponent *n* from the power law *P*_des_ ∝ *F*^*n*^, which is typical of DIMET processes, is plotted by the corresponding colored solid line. All of the *n* values are summarized in Table 1. For 0.75 and 0.33 ML, we have also studied the dependence of *P*_des_ upon the supercell size used in our calculations. As found by other authors, reaction properties can be sensitive to the size of the simulation cell. Good examples are the energy exchange in the scattering of CO on Au(111),^40^ the recombinative photodesorption probability of H_2_ on Ru(0001),^41^ and the equilibration of hot O atoms after O_2_ dissociation on Pd surfaces.^42^ In our case, the calculated exponents for 0.33 and 0.75 ML are rather insensitive to the increase of the supercell (see Table 1), although larger cells yield overall higher desorption probabilities, as shown in the left panel of Figure 2. The overall analysis together with the equal values obtained for 0.75 ML at 80 J/m^2^ with the 4 × 4 and 6 × 6 cells suggests that *P*_des_ differences start to be negligible when at least a 4 × 4 periodic box is used in the calculations. Hereinafter, we shall focus only on the biggest supercell theoretical results. For 0.60 ML, all of the dynamics simulations were performed in a large (5 × 2√3)rect cell.

**Figure 2:**
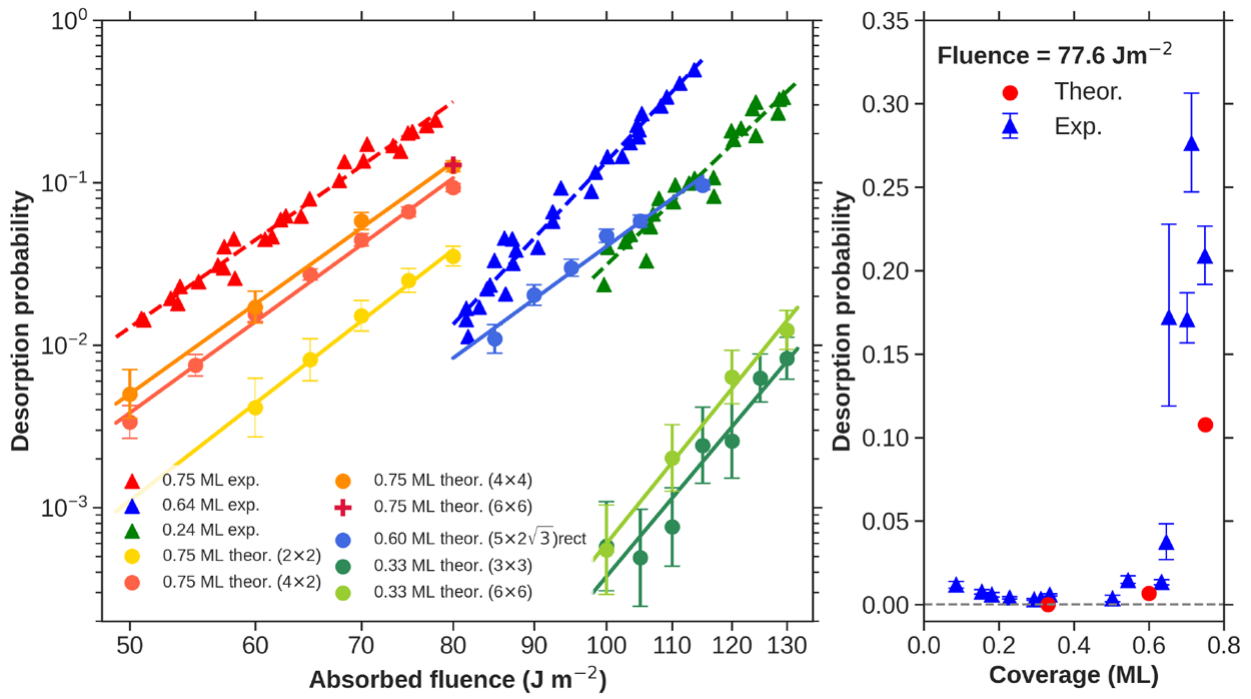
(Left) CO desorption probability as a function of absorbed fluence for different initial coverages. Reddish, bluish, and greenish full circles stand for (*T*_e_, *T*_l_)–MDEF theoretical results for 0.75, 0.60, and 0.33 ML coverages, respectively, with varying supercell models. Red, blue, and green triangles stand for experimental results taken from ref (28) for 0.75, 0.64, and 0.24 ML coverages, respectively. Full lines correspond to a log–linear model fit performed for each data set. (Right) CO desorption probability as a function of coverage for fixed absorbed fluence of 77.6 J/m^2^. Blue triangles stand for experimental results taken from ref (28). Red circles stand for theoretical results. Both sets of results come from log–linear extrapolations at the required fluence.

**Table 1:** Calculated (*T*_e_, *T*_l_)–MDEF Theoretical and Experimental^28^ Values of the Exponent *n* of the Power-Law *P*_des_ ∝ *F*^*n*^ for CO/Pd(111)-Induced Photodesorption for Various CO Coverages^a^

experiments^28^			
coverage	0.24 ML	0.64 ML	0.75 ML
*n*_exp_	9.3 ± 0.5	10.3 ± 0.4	6.9 ± 0.3

theory: (*T*_e_, *T*_l_)–MDEF			
coverage	0.33 ML	0.60 ML	0.75 ML
*n*_theo_	(3 × 3): 11.6 ± 1.5	(5 × 2√3)rect: 7.1 ± 0.5	(2 × 2): 7.6 ± 0.4
	(6 × 6): 12 ± 1		(4 × 2): 7.1 ± 0.3
			(4 × 4): 7.0 ± 0.3

a Cross products indicate the particular supercell used in theoretical calculations.

Table 1 shows that (*T*_e_, *T*_l_)–MDEF predicts a monotonic decrease in the power law exponents as coverage increases, i.e., *n*_theo_(0.33 ML) > *n*_theo_(0.60 ML) ≳ *n*_theo_(0.75 ML). As discussed elsewhere,^6,43^ the *n* value is expected to be roughly correlated with the effective number of energy level jumps that the adsorbate must experience to overcome the adsorption well. In this respect, the decremental sequence of (mean) CO desorption energies in the covered Pd(111) surface, i.e., *E*_d_(0.33 ML) > *E*_d_(0.60 ML) ≳ *E*_d_(0.75 ML) (see Table 2), explains the decreasing *n*_theo_ values obtained in our simulations as coverage increases. The same correlation between *E*_d_ and *n* was found theoretically for the site-specific photodesorption of O_2_ from Ag(110).^5^ With regard to the comparison to experiments,^28^Table 1 shows that the range of the theoretical power law exponents is in rather good agreement with the experimental power law exponents. Indeed, when both experiment and theory refer to the same coverage (i.e., 0.75 ML) the calculated *n*_theo_ (7.0 ± 0.3) is remarkably close to the experimental value *n*_exp_ (6.9 ± 0.3). The theoretical *n*_theo_ values for 0.60 and 0.33 ML, which have to be compared to the experimental results at 0.64 and 0.24 ML, respectively, differ from the experimental *n*_exp_ by ∼3 units. It is a reasonable difference considering how sensitive power law behaviors are to small variations in the system description. Qualitatively, however, *n*_exp_ shows a non-monotonic variation with coverage, where *n*_exp_(0.64 ML) ≳ *n*_exp_(0.24 ML) > *n*_exp_(0.75 ML). The latter could be a consequence of mobile CO that is speculated to exist at coverages below 0.33 ML,^28^ with an expected smaller desorption energy than at 0.33 ML.

**Table 2:** Site-Dependent Desorption Energies (eV) Calculated with the CO/Pd(111) EANN PES and DFT + vdW (*E*_des_^NN^/*E*_des_^DFT^) for Desorbing One CO from Pd(111) Covered with 0.33, 0.60, and 0.75 ML^a^

	top	bridge	hcp	fcc
0.33 ML				1.55/1.58
0.60 ML		0.85/0.95		0.78/0.87
0.75 ML	0.75/0.74		0.81/0.83	0.88/0.94

a The experimental desorption energies for 0.24, 0.64, and 0.75 ML (top site) were estimated as 1.38, 0.78, and 0.5 eV, respectively.^28^

With regard to the individual values of *P*_des_, it is apparent in Figure 2 that theoretical results lie below experimental measurements. To be more precise, theoretical 0.75 ML desorption probabilities underestimate experiments by a factor of ∼2, theoretical 0.60 ML by a factor of ∼3.5, and theoretical 0.33 ML by a factor of ∼32. The differences at 0.75 and 0.60 ML can be considered as minor in the context of these complex processes and attributable to certain overestimation of the underlying DFT desorption energies compared to the experimental measurements.^7^ In addition, part of the disagreement may come from differences in the compared coverages. This is specially true when comparing the theoretical results at 0.33 ML to the experimental measurements at 0.24 ML, because for coverages below 0.33 ML, it has been suggested by experiments that desorption is possibly enhanced by the formation of 0.33 ML islands and transient bridge-bound CO adsorbates.^28,44^ Such processes would require much bigger supercells to be modeled, which are still unfeasible for our (*T*_e_, *T*_l_)–MDEF simulations and out of the scope of this work. Nonetheless, the three simulated coverages do already serve to study the coverage dependence as done in experiments.^28^ Remarkably, our results reproduce the strikingly strong dependence of the desorption yield upon the coverage measured for this system. This is shown in the right panel of Figure 2, where we reproduce the extrapolated experimental desorption probabilities at *F* = 77.6 J/m^2^ ^28^ as a function of coverage (blue triangles) together with our theoretical results (red dots), which are extrapolated for the same fluence as done in ref (28). Both theory and experiments show an increase of reaction probabilities with coverage with a sharp enhancement when passing from intermediate (∼0.5 ML) to high (∼0.75 ML) coverages. As argued above, such a behavior is attributable to the dependence of CO desorption energies upon coverage.

A better understanding of the electron and phonon contributions to the CO photodesorption process, which could not be discerned by the 2PC measurements,^28^ as well as their dependence upon coverage can now be obtained by comparing in Figure 3 the (*T*_e_, *T*_l_)–MDEF results (full lines) to those obtained with *T*_e_–MDEF (dotted lines), which only include the effect of hot electrons, and with *T*_l_–MDEF (dashed lines), which only include the effect of the electron-induced hot lattice. The figure shows the temporal profiles of CO desorption probabilities obtained for the lowest (0.33 ML, right panels) and highest (0.75 ML, left panel) CO coverages for a set of different laser fluences that encompass the range studied in Figure 2, namely, 60, 70, and 80 J/m^2^ for 0.75 ML and 100 and 130 J/m^2^ for 0.33 ML. The origin of time coincides with the instant at which the temporal profile of the incoming laser pulse reaches its maximum. For both coverages, the (*T*_e_, *T*_l_)–MDEF desorption probabilities are higher than those of *T*_e_–MDEF and *T*_l_–MDEF during the whole simulation time. This means that neither hot electrons nor hot surface phonons alone, taken as individual mechanisms, are as efficient as the combined process to induce desorption. Furthermore, if we focus on the first 10–20 ps of the dynamics (see insets in Figure 3), the (*T*_e_, *T*_l_)–MDEF desorption consistently starts before the simulated electron-only and phonon-only desorption. This result highlights that the conjoined contribution of both channels enhances a quick and pronounced desorption response on the irradiated system. Thus, it corroborates the synergistic effect that hot electrons and phonons produce on the CO photodesorption on Pd found in previous AIMDEF studies^7^ and extends its consequences to longer time regimes (up to 100 ps) that are unreachable with the mentioned (*T*_e_, *T*_l_)–AIMDEF simulations.

**Figure 3:**
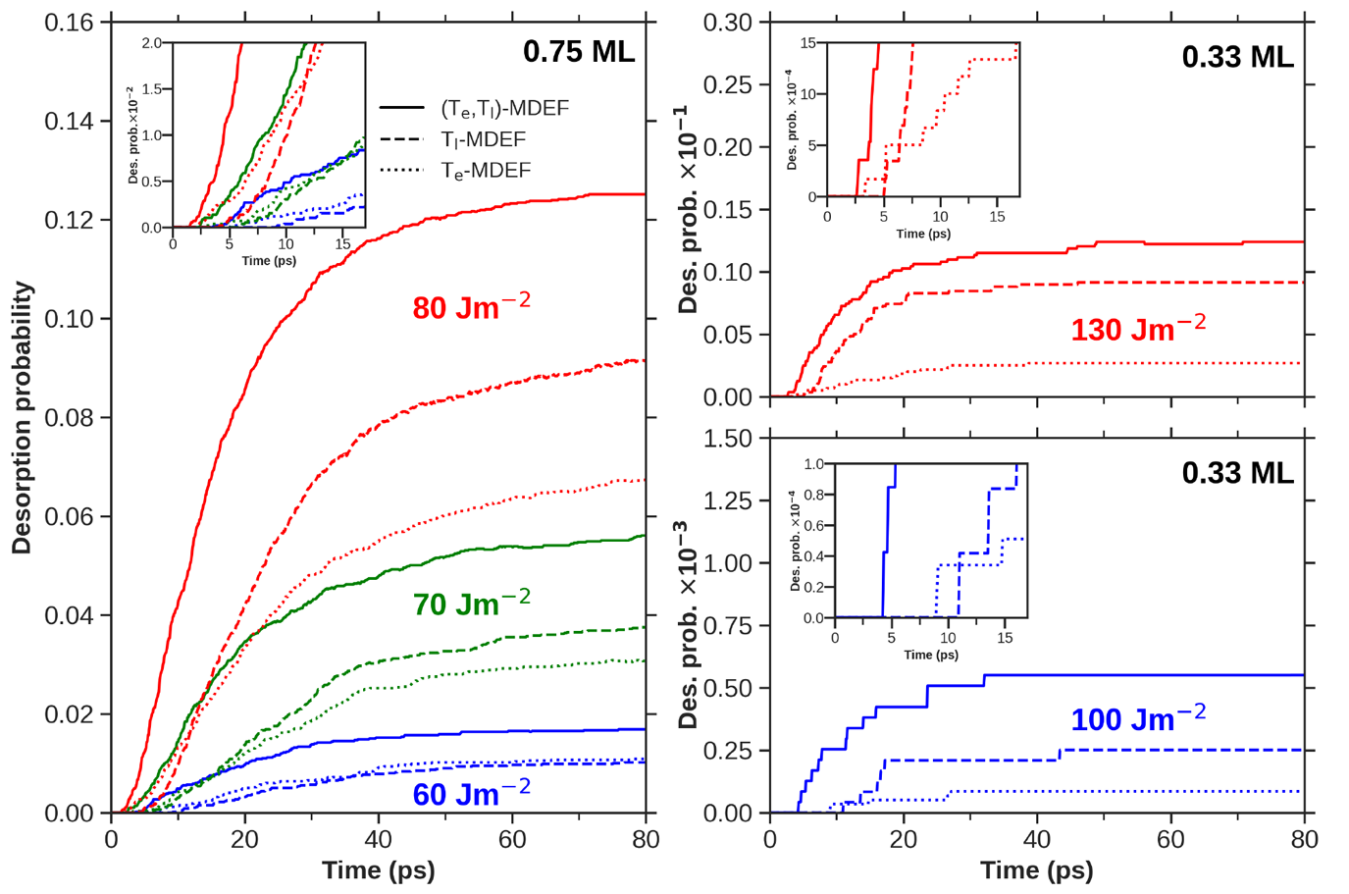
Mean CO desorption probability as a function of time, with 100 fs resolution. (*T*_e_, *T*_l_)–MDEF results are shown in full lines; *T*_e_–MDEF results are shown in dotted lines; and *T*_l_–MDEF results are shown in dashed lines. (Left) MDEF results obtained for the 0.75 ML coverage and fluences 60 J/m^2^ (blue), 70 J/m^2^ (green), and 80 J/m^2^ (red). (Right) MDEF results obtained for the 0.33 ML coverage and fluences 100 J/m^2^ (blue) and 130 J/m^2^ (red). Insets correspond to zooms into the early stages of the photodesorption process.

Focusing now on the *T*_e_–MDEF and *T*_l_–MDEF photodesorption probabilities, we obtain that the *T*_e_–MDEF desorption is always initiated before that of *T*_l_–MDEF (see insets in Figure 3). This is a consequence of the high temperatures that hot electrons reach during the first picoseconds of the dynamics according to TTM (see Figure S2 of the Supporting Information^33^). However, for laser fluences higher than 60 J/m^2^ in the 0.75 ML coverage and for the two (high) fluences calculated in the 0.33 ML coverage, the *T*_l_–MDEF desorption probability surpasses the *T*_e_–MDEF values by several picoseconds after the desorption starts. The crossing occurs at an earlier instant the higher the laser fluence is. Also, the contribution of phonons to the final desorption probability (e.g., values at *t* = 80 ps) becomes increasingly important as *F* increases. Among the calculated cases, it is only for 60 J/m^2^ and 0.75 ML that both simulations, *T*_e_–MDEF and *T*_l_–MDEF, predict almost equal values (see the left panel of Figure 3). Putting aside the synergistic effect that hot electrons and phonons have on desorption, our simulations show that, although the hot electron mechanism may dominate during the first picoseconds after laser irradiation, the action of hot surface phonons is by no means negligible and can even dominate CO photodesorption at high laser fluences as a result of the high transient lattice temperature reached in Pd(111). In this respect, Pd is characterized by a significantly high electron–phonon energy exchange coupling constant (around 8.95 × 10^17^ W K^–1^ m^–3^ ^45^), and therefore, the TTM-predicted maximum lattice temperatures on the surface for 60, 70, 80, 100, and 130 J/m^2^ fluences are around 638, 718, 797, 952, and 1180 K, respectively (Figure S2 of the Supporting Information^33^).

The (*T*_e_, *T*_l_)–MDEF simulations provide direct information about the CO photodesorption dynamics that cannot be extracted from the usual kinetic models. In agreement with previous (*T*_e_, *T*_l_)–AIMDEF,^7^ the present simulations show that CO moves all over the surface. As a representative example of the high CO mobility, we show in Figure S6 of the Supporting Information for 0.75 ML and *F* = 80 J/m^2^ the center of mass position over the surface of all desorbing CO that, at any instant, is located at a height *Z*_CO_ ± δ*Z*_CO_. Note that initially the adsorbates are well-located around top, hcp, and fcc sites (blue dots), but the plot at *Z*_CO_ = 2.5 ± 0.5 Å is completely covered, as a clear demonstration of the high CO mobility. An interesting finding that could not be observed during the first 3.5 ps that lasted the AIMDEF simulations is the eventual trapping of desorbing CO in the physisorption region. Figure 4 shows for each coverage and two distinct fluences the time evolution of the normalized probability density of finding the CO center of mass height *Z*_CO_ at a given distance from the mean position of the Pd topmost layer *Z*_surf_. In all cases, it is apparent that CO molecules can either desorb directly (some examples in white lines) or become transiently trapped in the region around 5–7.5 Å from the surface (2.5–5 Å from the adlayer) for several picoseconds before desorption (some examples in cyan lines). Interestingly, the analysis of the polar angle distribution of the center of mass velocities shows that, in the direct desorption events, the molecules do not necessarily desorb along the surface normal but at different off-normal angles (see Figure S7 of the Supporting Information^33^).

**Figure 4:**
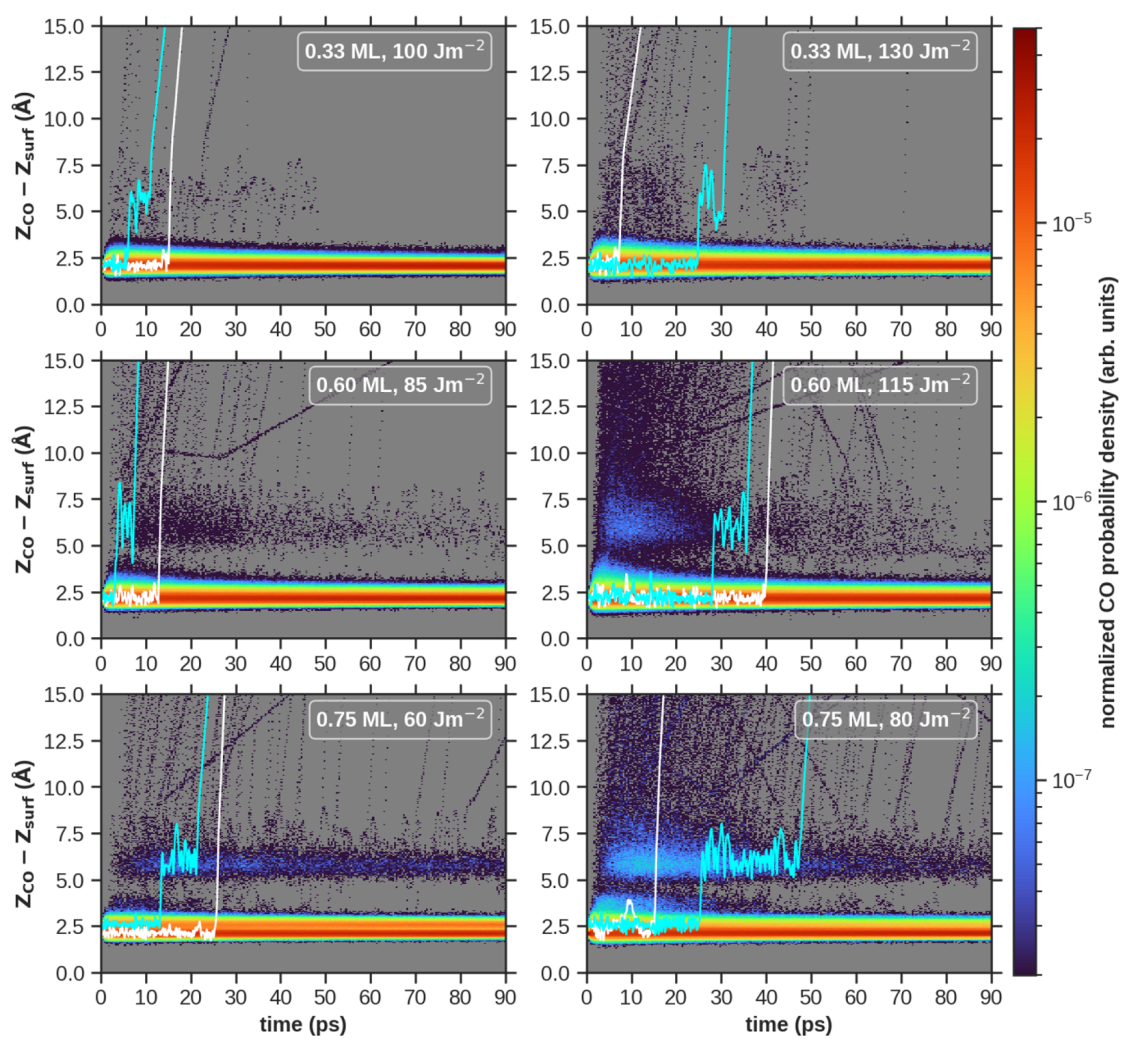
Probability density map of the CO center of mass height (*Z*_CO_) from the topmost Pd layer (*Z*_surf_) as a function of time. From top to bottom, results are shown in increasing coverage order: 0.33 ML (top), 0.60 ML (middle), and 0.75 ML (bottom). From left to right, results are shown in increasing absorbed fluence order, which varies between coverages: 100 J/m^2^ (top left), 130 J/m^2^ (top right), 85 J/m^2^ (middle left), 115 J/m^2^ (middle right), 60 J/m^2^ (bottom left), and 80 J/m^2^ (bottom right). White and cyan full lines mark examples of direct CO desorption and desorption after transient trapping, respectively.

In conclusion, we studied the coverage dependence of the femtosecond laser-induced desorption of CO from a Pd(111) surface. To this aim, we have performed MD simulation using an improved implementation of the (*T*_e_, *T*_l_)–MDEF model in which all C, O, and moving Pd atoms are coupled to the laser excited electronic system via a Langevin thermostat with a time-dependent electronic temperature *T*_e_(*t*). The adiabatic forces are obtained from a multicoverage NN PES that was trained with DFT data calculated for 0.75 and 0.33 ML CO coverages. The high quality of the NN PES and its transferability to treat other coverages are demonstrated, among other things, by the fact that it is able to predict the DFT equilibrium structure for the 0.60 ML CO coverage even if the NN PES was not trained with configurations of this coverage. The results of our dynamics agree well with existing experiments regarding the fluence dependence of the desorption yield for the different coverages and reproduce the experimentally observed strong dependence of the desorption yield upon the CO surface coverage, in particular, its sharp increase from intermediate to high coverages. All of these results are explained in terms of the coverage decreasing desorption energies.

By performing simulations in which only the electronic or phononic channels are included, we demonstrate that both channels play important roles in the desorption process. We also find that the relative importance of the phononic channel increases for large laser fluences and low CO surface coverages. Finally, our simulations permit us to gain information on the characteristics of the desorption process and reveal that, although important amounts of molecules desorb directly, interestingly, some of them are trapped for several picoseconds before desorption in the physisorption region. This dynamical trapping mechanism is more important at large coverages, which leads us to infer that it is governed by the attractive van der Waals CO–CO forces.

## Supporting Information Available

Further information on the (*T*_e_, *T*_l_)–MDEF method, the multicoverage EANN PES, and the photoinduced CO desorption dynamics (PDF)
