# Supplementary material for: Multicoverage Study of Femtosecond Laser-Induced Desorption of CO from Pd(111)
Source: J Phys Chem Lett. 2024 Feb 28;15(9):2587–94. doi: 10.1021/acs.jpclett.4c00026 (PMC10926157; doi:10.1021/acs.jpclett.4c00026)
Supplement: Supplementary file 1 — jz4c00026_si_001.pdf [file jz4c00026_si_001.pdf]

Supporting Information: Multicoverage Study of Femtosecond Laser Induced Desorption of CO from Pd(111)

Alberto S. Muzas,[*,†] Alfredo Serrano Jiménez,[*,†] Yaolong Zhang,[‡] Bin Jiang,[*,‡] J. Iñaki Juaristi,[*,†,¶,§] and Maite Alducin[*,†,¶]

† *Centro de Física de Materiales CFM/MPC (CSIC-UPV/EHU), Paseo Manuel de Lardizabal 5, 20018 Donostia-San Sebastián, Spain*

‡*Hefei National Laboratory for Physical Science at the Microscale, Key Laboratory of Surface and Interface Chemistry and Energy Catalysis of Anhui Higher Education Institutes, Department of Chemical Physics, University of Science and Technology of China, Hefei, Anhui 230026, China*

¶ *Donostia International Physics Center (DIPC),Paseo Manuel de Lardizabal 4, 20018 Donostia-San Sebastián, Spain*

§ *Departamento de Polímeros y Materiales Avanzados: Física, Química y Tecnología, Facultad de Químicas (UPV/EHU), Apartado 1072, 20080 Donostia-San Sebastián, Spain*

E-mail: alberto.muzas@uam.es; aserrano030@ikasle.ehu.eus; bjiangch@ustc.edu.cn; josebainaki.juaristi@ehu.eus; maite.alducin@ehu.eus

DFT Computational Details

As described in the main text, the accuracy of the CO/Pd(111) EANN-PES for the (untrained) 0.60 ML coverage was tested against density functional theory (DFT) calculations that basically use the same computational settings of the (T_e, T_l)-AIMDEF simulations presented in ref S1 for 0.75 and 0.33 ML coverages. Specifically, all DFT calculations are performed with VASP[S2,S3] (version 5.4) using the vdW-DF exchange-correlation functional proposed by Dion *et al.*[S4] The electron-core interaction is treated with the projector augmented-wave (PAW) method[S5] using the VASP PAW potentials[S6] for C, O, and Pd that have four, six, and ten valence electrons, respectively. Additional input parameters are: an energy cutoff of 400 eV for the plane-wave basis set, a Methfessel and Paxton first-order broadening scheme of 0.1 eV for electron fractional occupancies,[S7] an energy criteria for total energy self-consistency of 10^{-6} eV, and a Γ-centered 3×6×1 Monkhorst-Pack grid of special **k**-points.[S8] The supercell consists of a $(5 \times 2\sqrt{3})$rect surface cell (blue rectangle in Figure S1) and a 4 layer-slab separated by 8 layers of vacuum.

The desorption energy E_{des} provided in Table 1 of the main text for different CO adsorption sites in each coverage corresponds to the energy required to desorb a single CO adsorbate. Each energy is calculated as the difference between the energy of the optimized structure in which one CO is oriented parallel to the surface and moved from the surface adlayer to roughly a midway position between the CO adlayer and the bottom of the periodic Pd(111) surface and the energy of the optimized CO/Pd(111) covered surface.

CO/Pd(111) Model Details

Figure S1 shows all the supercells and coverages that have been used in the present work. The structural CO patterns shown on each panel are the local minima found with the trained EANN-PES, source of the adiabatic forces in (T_e, T_l)-MDEF calculations. For 0.33 ML and 0.75 ML of CO coverage (top-left and top-right panels, respectively), the plotted struc-

tures are also the global minima, which coincides with previous experimental reports.[S9] For 0.60 ML, our EANN-PES predicts a complex $(5 \times 2\sqrt{3})$rect-12CO structure (bottom right) that is slightly more stable according to both our EANN-PES and DFT+vdW-DF (see discussion in main text) than the c$(5 \times \sqrt{3})$rect-3CO arrangement (bottom left) reported for this coverage by other authors.[S9,S10]

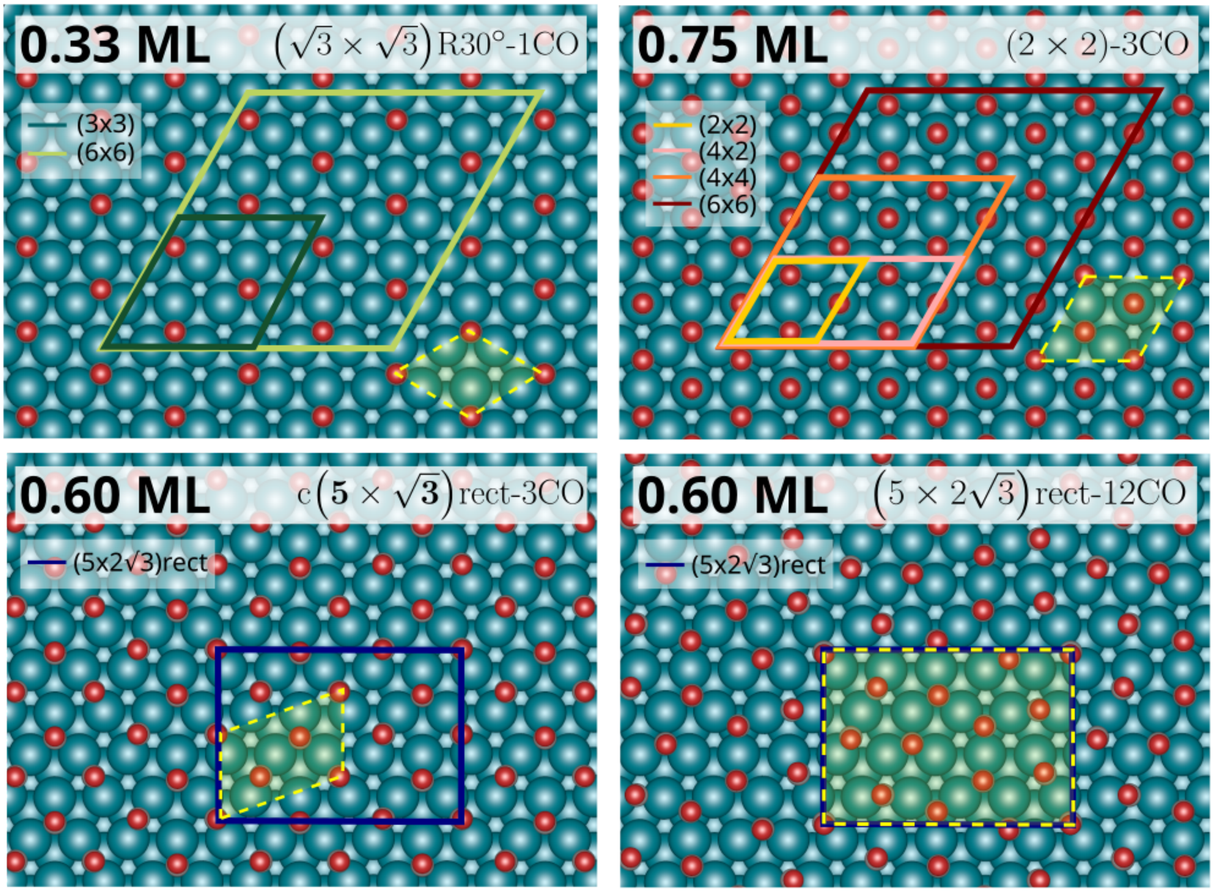

Figure S1: Top view of minimum energy configurations calculated with the EANN-PES for 0.33 ML (top left), 0.75 ML (top right), and 0.60 ML (bottom left and right) of CO coverage. In the latter case, the bottom left structure corresponds to a local minimum higher in energy than the bottom right structure. The actual supercells used in the (T_e, T_l)-MDEF simulations are represented in full lines. Yellow dashed lines and shaded areas show the smallest pattern that is repeated within supercells.

Multicoverage EANN Training and Quality Assessment

In order to perform the theoretical calculations, the preliminary step consists on having an appropriate PES for different CO/Pd(111) coverages. Its training is performed with the embedded atom neural network (EANN) method,[S11] already used to generate a PES for 0.75 ML CO/Pd(111) from previous (T_e, T_l)-AIMDEF data.[S12] There is also (T_e, T_l)-AIMDEF available data for 0.33 ML CO/Pd(111) as well as T_e-AIMDEF data for both coverages.[S1] Recall that in the (T_e, T_l)-AIMDEF simulations the adsorbate atoms follow a Langevin dynamics, in which the friction and random forces account for the effect of the hot electrons, and the Pd surface atoms are connected to a Nosé-Hoover thermostat that assures the lattice temperature predicted by TTM, whereas in T_e-AIMDEF the adsorbate atoms are allowed to move following the Langevin dynamics as before but all Pd surface atoms are frozen. Data from these four pools is used for training the aimed multicoverage PES. However, since the supercell employed in the AIMDEF simulations for each coverage was different, there is an energy mismatch between the 0.33 ML and 0.75 ML DFT total energies. To overcome this issue, we use the 0.75 ML CO/Pd(111) EANN-PES of ref S12 to predict the energies of the 350 000 (T_e, T_l)-AIMDEF configurations that are available in the case of 0.33 ML. A linear fit in which the slope is taken as $m = 1$ is then applied to the predicted energies to correct the offset value. As a result, we calculate an offset energy of $b = -22.04651$ eV that is subsequently added to the 0.33 ML AIMDEF energies.

Once the energy mismatch between the two coverages is solved, we train a PES containing two hidden layers of 60 nodes per layer, having as input 60 embedded density descriptors per atom element (15 Gaussian-type orbitals (GTOs) for allowed angular momentum values of $L = 0 - 3$).[S11] Remaining EANN-model parameters on GTO geometry, cutoff radius, and energy and force weights for the cost function are chosen as in ref S12. The first training involved 34 283 configurations. 90% corresponds to (T_e, T_l)-AIMDEF data that consist of the 15 883 configurations used in ref S12 to obtain the 0.75 ML CO/Pd(111) EANN-PES plus new 15 000 configurations from 0.33 ML. The remaining 10% comes from

the $T_{\rm e}$-AIMDEF pool and includes 1700 configurations from each coverage. Selection of these initial configurations that conform the initial training set is done according to the energy representativity criteria described in ref S12. Among the training configurations, 90% of the pool was randomly chosen for training, whilst the remaining 10% is used to check that the PES has not been overfitted. Five PESs are trained in this first leg, using an extreme machine learning Levenberg-Marquardt algorithm for the error minimization .[S13] The EANN convergence is very fast, involving no more than 45 iterations for these PESs, and yields for the energy root-mean-square-error (RMSE) values of 0.55–0.65 meV and 0.84–0.92 meV per moving atom for the training and validation data sets, respectively. Notice that, as the contribution of each coverage to the total training set is about 50%, the mentioned error values per moving atom are calculated by dividing the total error by 34.5, i.e., the average of the moving atoms of both coverages (33 for 0.33 ML and 36 for 0.75 ML).

These starting PESs are validated afterwards against four predict sets of 140 766 (0.33 ML $T_{\rm e}$-AIMDEF), 116 297 (0.33 ML $(T_{\rm e}, T_{\rm l})$-AIMDEF), 140 692 (0.75 ML $T_{\rm e}$-AIMDEF) and 87 382 (0.75 ML $(T_{\rm e}, T_{\rm l})$-AIMDEF) configurations that were absent from the training input data. Predictions are performed separately for each data pool due to the differences in the amount of total and moving atoms in each set. We study the 10 absolute-valued maximum force errors for each moving atom and component, being each force error defined as $|\Delta F_i| = |F_i^{\rm EANN} - F_i^{\rm DFT}|,\ i = x, y, z,$ as described in ref S12, and choose the PES with the overall smaller error values. In this case, these maximum-valued force errors correspond to 945 (0.33 ML $T_{\rm e}$-AIMDEF), 863 (0.33 ML $(T_{\rm e}, T_{\rm l})$-AIMDEF), 877 (0.75 ML $T_{\rm e}$-AIMDEF) and 1 004 (0.75 ML $(T_{\rm e}, T_{\rm l})$-AIMDEF) configurations, which after their incorporation to the initial training pool raise the number of configurations in the training set to 37 972. Finally, five new PESs are retrained starting from the previously selected PES. Then, comparison of maximum absolute-valued error forces for each data set leads us to choose our final PES to perform the MDEF simulations. Using the aforementioned criterium for the amount of moving atoms, the latter PES has total energy RMSE values per moving atom of 0.64 meV

and 0.80 meV for the training and validation sets, respectively.

Table S1: Maximum absolute-valued errors and RMSEs per moving atom in energies and in atomic forces predicted by the final multicoverage EANN-PES. For 0.33 ML and 0.75 ML, we provide separately the error obtained in configurations extracted from the T_e-AIMDEF and $(T_\mathrm{e}, T_\mathrm{l})$-AIMDEF simulations. Errors for 0.60 ML are evaluated from a set of configurations that were extracted randomly from the $(T_\mathrm{e}, T_\mathrm{l})$-MDEF simulations. Maximum errors are defined as $|\Delta Y|_\mathrm{max} = \left|Y^\mathrm{EANN} - Y^\mathrm{DFT}\right|_\mathrm{max}$, with $Y = E,\ F_\beta\ (\beta = x, y, z)$. Total number of moving atoms N_mov is 33 for 0.33 ML, 36 for 0.75 ML, and 84 for 0.60 ML.

		T_e-AIMDEF		$(T_\mathrm{e}, T_\mathrm{l})$-AIMDEF		$(T_\mathrm{e}, T_\mathrm{l})$-MDEF
		**0.33 ML**	**0.75 ML**	**0.33 ML**	**0.75 ML**	**0.60 ML**
E/N_mov (meV)	Max. error	1.407	2.441	6.093	9.311	5.532
	RMSE	0.210	0.312	1.014	0.954	1.205
F_x (eV/Å)	Max. error	0.1735	0.2331	0.9800	0.5073	0.3537
	RMSE	0.0146	0.0167	0.0488	0.0536	0.0489
F_y (eV/Å)	Max. error	0.1617	0.3138	0.5683	0.5045	0.3822
	RMSE	0.0144	0.0161	0.0488	0.0519	0.0489
F_z (eV/Å)	Max. error	0.3067	0.3492	0.6356	0.5938	0.4402
	RMSE	0.0187	0.0230	0.0498	0.0589	0.0670

This final PES behaves solidly when evaluating the energies and forces of the 485 137 predict set configurations in each of the four possible cases (i.e., T_e-AIMDEF for 0.33 ML and 0.75 ML and $(T_\mathrm{e}, T_\mathrm{l})$-AIMDEF for 0.33 ML and 0.75 ML), as confirmed by the maximum errors in absolute value and RMSEs of energies and forces summarized in Table S1. Notice that, in the table, maximum absolute-valued errors and RMSEs for the energy are computed using all configurations in each pool. For each force coordinate, instead, computation is performed for the moving atoms, leaving aside the Pd atoms that are kept frozen during the simulations. In Figure S2 we also plot the usual comparison of the EANN PES energies against the corresponding DFT energies. The errors shown in Table S1 for 0.60 ML (i.e., the coverage for which the EANN-PES was not trained) are evaluated using a set of 1446 configurations that have been extracted randomly from the $(T_\mathrm{e}, T_\mathrm{l})$-MDEF simulations performed in this work at different fluences, $F = 85.0, 95.0$, and 115 J/m^2.

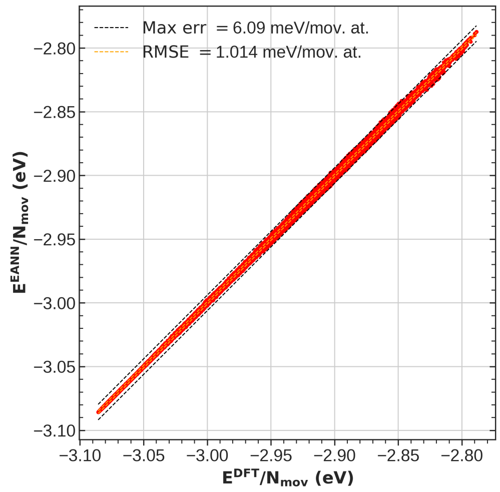

(a) Coverage: 0.33 ML

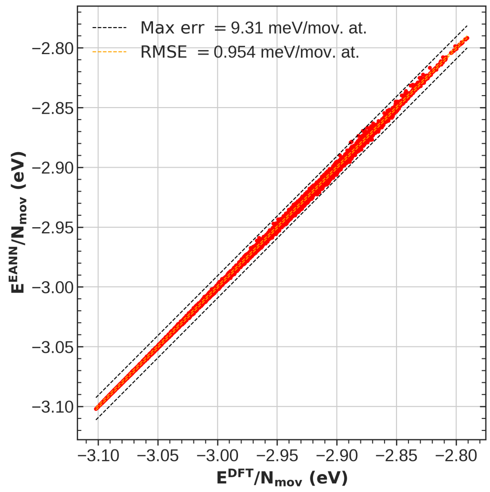

(b) Coverage: 0.75 ML

Figure S2: Comparison of the interaction energy (as provided by the VASP code) per moving atom computed with the (final) multicoverage CO/Pd(111) EANN-PES and the corresponding DFT values for the predict set extracted from (T_e, T_l)-AIMDEF.

Minimum energy paths under equilibrium conditions

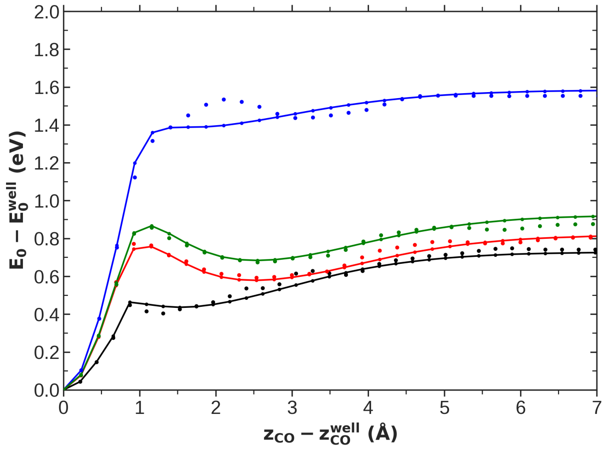

Figure S3: Potential energy as a function of the CO center-of-mass height Z_{CO} for dsorbing one CO from 0.33 ML and different adsorption sites in 0.75 ML. Energies calculated in ref S1 with DFT+vdW-DF (solid lines) and with EANN PES (symbols). In each curve the energy and height are referred to the values obtained at the bottom of the corresponding well.

Figure S3 shows the minimum energy paths calculated with DFT+vdWDF in ref S1 for 0.33 ML and 0.75 ML. Each curve corresponds to desorption of one CO along the surface

normal. For comparison the EANN PES values are also included. The agreement is rather good considering that no information on desorption under equilibrium conditions was used for training.

Photoinduced Desorption Dynamics: $(T_\mathrm{e}, T_\mathrm{l})$-MDEF Simulations

As reviewed in the main text, the perturbation created by femtosecond laser pulses in metal surfaces is commonly described with the two-temperature model (TTM) in terms of two (linearly) coupled thermal baths that are respectively characterized by two distinct time-dependent temperatures $T_\mathrm{e}(t)$ and $T_\mathrm{l}(t)$.[S14] The former represents the electron excitations created directly by the laser pulse, while the latter the phonon excitations induced by the excited electrons trough the electron-phonon coupling. As explained elsewhere,[S15,S16] the diameter of the laser beam and the time scale of interest justify to assume a constant laser fluence in the plane parallel to the metal surface and neglecting lattice thermal diffusion into the bulk. Under these assumptions, the TTM equations read,

$$\begin{aligned} C_\mathrm{e}\frac{\partial T_\mathrm{e}}{\partial t} &= \frac{\partial}{\partial z}\left(\kappa_\mathrm{e}\frac{\partial T_\mathrm{e}}{\partial z}\right) - G(T_\mathrm{e}-T_\mathrm{l}) + S(z,t) \\ C_\mathrm{l}\frac{\partial T_\mathrm{l}}{\partial t} &= G(T_\mathrm{e}-T_\mathrm{l}), \end{aligned} \tag{S1}$$

where C_e and C_l are the electron and lattice heat capacities, respectively; κ_e is the thermal conductivity of the electron subsystem; G is the electron-phonon energy exchange coupling constant; z is the distance from the surface plane; and $S(z,t)$ is the absorbed laser power per unit volume that depends on the shape, wavelength, duration, and fluence of the applied pulse.

Figure S4 shows the electron T_e (left panel, full lines) and lattice T_l (right panel, full lines) temperatures of Pd(111) initially at 90 K calculated with the TTM for a 780 nm and 130 fs

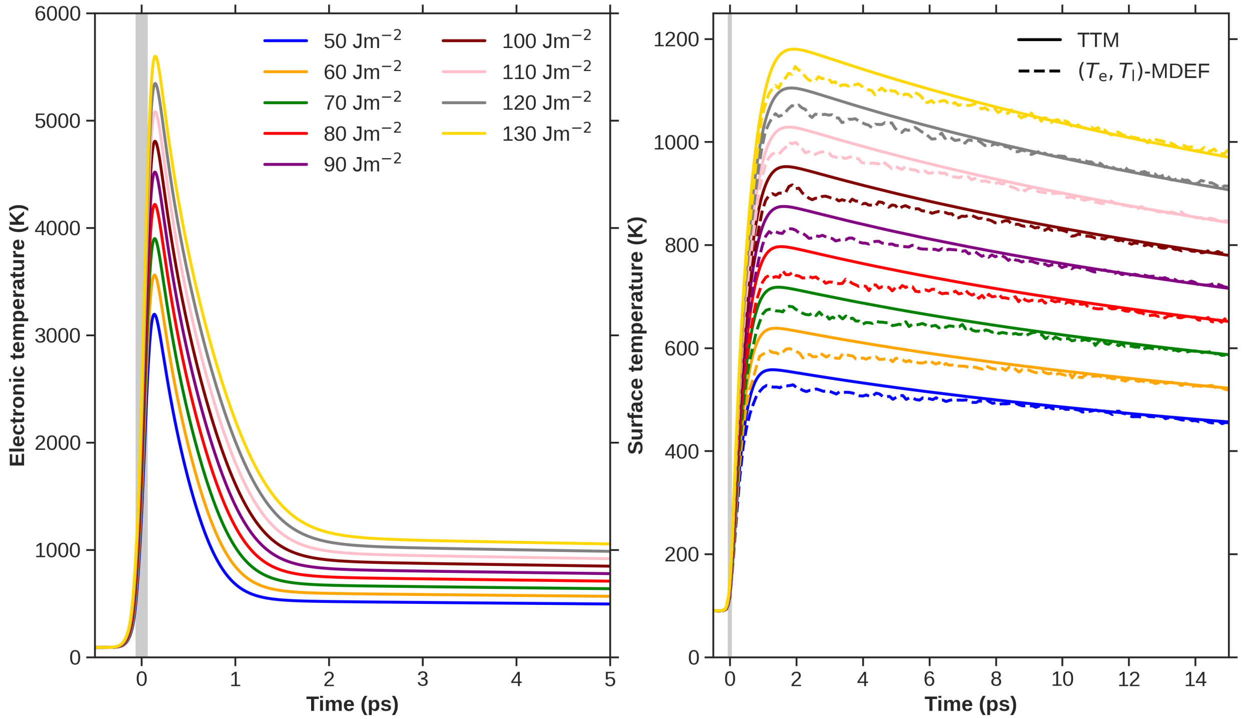

Figure S4: Electronic (left panel) and surface (right panel) temperature profiles of Pd calculated with the TTM (full lines) for different laser fluences covering the range studied in the main text. Dashed lines correspond to the temperature average of the three topmost mobile Pd layers in $(T_\mathrm{e}, T_\mathrm{l})$-MDEF calculations. The maximum intensity of the incoming laser pulse is reached at time zero. Gray shaded area corresponds to the full width at half maximum of the laser pulse.

sech^2 laser pulse excitation and various laser fluences. These are the experimental conditions used in ref S9. The specific expressions describing the experimental laser pulse shape and the macroscopic thermal coefficients $C_\mathrm{l}(T_\mathrm{l})$, $\kappa_\mathrm{e}(T_\mathrm{e}, T_\mathrm{l})$, and G for the Pd(111) surface are taken from ref S17. C_e is defined as $C_\mathrm{e}(T_\mathrm{e}) = T_\mathrm{e}\gamma(T_\mathrm{e})$ where γ has been fitted to previously published measurements (see Figure S5) according to formula:

$$\gamma(T) = a_0 + a_1 \exp\left(-\frac{T - T_1}{\sigma_1}\right) + a_2 \cosh^{-2}\left(\frac{T - T_2}{\sigma_2}\right) + a_3 \cosh^{-2}\left(\frac{T - T_3}{\sigma_3}\right), \qquad \text{(S2)}$$

where fitting parameters a_i, T_i, and σ_i can be found in Table S2.

Once T_e is known, the effect of the excited electrons in the dynamics of each atom

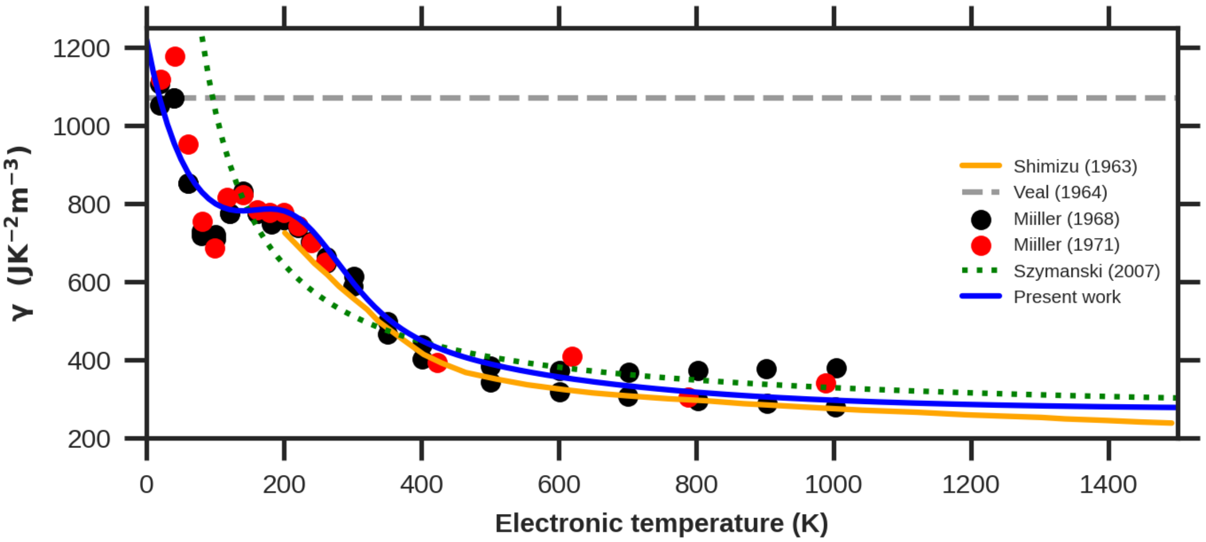

Figure S5: Electronic heat capacity coefficient γ in palladium as a function of electronic temperature. $\gamma(T_\mathrm{e})$ used in our calculations (blue line), fitted to experimental data Shimizu (1963)[S18] (orange line), Miiller (1968)[S19] (black dots), and Miiller (1971)[S20] (red dots). $\gamma(T_\mathrm{e})$ derived from a fit in previous works Szymanski (2007)[S17] (dotted green line). $\gamma(0)$ derived from heat capacity low temperature measurements in Veal (1964)[S21] (gray dashed line).

conforming the adlayer and metal surface is described trough the following Langevin equation

$$m_i \frac{d^2\mathbf{r}_i}{dt^2} = -\nabla_{\mathbf{r}_i} V(\mathbf{r}_1, ..., \mathbf{r}_N) - \eta_{e,i}(\mathbf{r}_i)\frac{d\mathbf{r}_i}{dt} + \mathbf{R}_{e,i}[T_e(t), \eta_{e,i}(\mathbf{r}_i)]\,, \tag{S3}$$

where m_i, $\mathbf{r}_i$, and $\eta_{e,i}$ are the mass, position vector, and electronic friction coefficient of the ith atom in the system. The first term in the right hand side of the equation is the adiabatic force that depends on the position of all (adsorbates and surface) atoms. The second and third terms are the electronic friction and electronic stochastic forces, respectively, that describe the effect of the electronic excitations and deexcitations on the adsorbate and lattice dynamics. Both forces are related through the fluctuation-dissipation theorem, being $\mathbf{R}_{e,i}$ modeled by a Gaussian white noise with variance

$$\mathrm{Var}[\mathbf{R}_{e,i}(T_e, \eta_{e,i})] = \frac{2k_B T_e(t)\eta_{e,i}(\mathbf{r}_i)}{\Delta t}\,, \tag{S4}$$

Table S2: Fitting parameters used for $\gamma(T_e)$ in Equation S2.

Parameter	Value	Units
a_0	273	$JK^{-2}m^{-3}$
a_1	2 702	$JK^{-2}m^{-3}$
a_2	214	$JK^{-2}m^{-3}$
a_3	98 180	$JK^{-2}m^{-3}$
T_1	-96	K
T_2	213	K
T_3	-2 085	K
σ_1	50	K
σ_2	103	K
σ_3	634	K

where k_B and Δt are the Boltzmann constant and the time-integration step, respectively.

The electronic friction coefficient for each atom in the adlayer (C and O) is calculated within the local density friction approximation (LDFA),[S22,S23] which requires an on the fly evaluation of the electronic density generated by the surrounding surface atoms as detailed in Serrano *et al.*[S12] Each Pd friction coefficient has been computed by assuming linear dependence with respect to its embedding electronic density, i.e. $\eta_i^{\mathrm{Pd}} \propto \rho(\mathbf{r}_i)$, which can be expressed as:

$$\eta_i^{\mathrm{Pd}}(r) = \left(\frac{r_0}{r}\right)^3 \eta^{\mathrm{Pd}}(r_0), \tag{S5}$$

where r is the Wigner-Seitz radius associated to the embedding electronic density $\rho(\mathbf{r}_i)$ of palladium atom i, and r_0 is a fixed Weigner-Seitz radius for which $\eta^{\mathrm{Pd}}(r_0)$ is calculated. We have chosen $\eta^{\mathrm{Pd}}(r_0)$ to be consistent with the electron-phonon energy exchange constant G used in the TTM. Following the same reasoning as in Duffy *et al.*,[S24] the latter quantities are related by the following simple expression:

$$\eta^{\mathrm{Pd}}(r_0) \approx \frac{m_{\mathrm{Pd}}}{3k_{\mathrm{B}}d_{\mathrm{Pd}}} G = 1.6223 \text{ a.u.}, \tag{S6}$$

where m_{Pd} and d_{Pd} are the atomic mass and the atomic density (here taken as 4 atoms per 64 Å^3) of Pd, respectively. Given that G is a bulk property of Pd, we have conveniently set

r_0 as the Weigner-Seitz radius of the embedding electronic density of a Pd atom in the bulk as predicted from our model, i.e., $r_0 = 4.38$ a.u. As shown in the right panel of Figure S4, the heating of mobile Pd atoms during $(T_\mathrm{e}, T_\mathrm{l})$-MDEF trajectories (dashed lines) closely follows surface lattice temperatures predicted by the TTM (full lines) during the early stages of the photodesorption process (from 0 to 12 ps). And at longer times, the agreement is excellent, validating the approximations taken in the evaluation of palladium friction coefficients.

Calculation of Observables

A CO molecule is counted as desorbed, if its center of mass height Z_CO measured from the mean position of the Pd topmost layer Z_surf is greater than 10 Å. The CO desorption probability is calculated for each coverage and fluence as

$$P_\mathrm{des} = \frac{N_\mathrm{des}}{N_\mathrm{t} N_\mathrm{CO}} \tag{S7}$$

where N_des is the number of desorbed CO molecules, N_t is the total number of MDEF trajectories, and N_CO is the number of CO molecules in the simulation cell. Depending on which laser fluence, coverage, and cell size were used in the simulation, the total number of trajectories N_t varied within the range 500–5000 to assure that the corresponding P_des value is accurate enough (error bars indicated in Figure 2 in the main text).

The time-resolved desorption probabilities in Figure 3 of the main text are calculated at each integration step t using as N_des in equation S7, the total number of CO molecules that are desorbed within the time interval $[0, t]$.

Additional details of the adsorbate dynamics

As remarked in the main text the laser pulse creates a highly excited environment that includes not only the initial hot electrons but also the (electron-induced) hot Pd and CO lattices (see for instance in Figure S4 that the Pd(111) temperatures may vary in the range

$\sim$400–1200 K, depending on fluence). In particular, the CO molecules become highly vibrationally excited, tilting much while diffusing on the surface plane and sometimes along the surface normal. As an example of the high CO mobility induced by the laser pulse, Figure S6 shows for 0.75 ML and an absorbed laser fluence $F = 80$ J/m^2 the $(X_{\mathrm{CO}}, Y_{\mathrm{CO}})$ center of mass coordinates of the desorbing CO at different distances from the surface $Z_{\mathrm{CO}} \pm 0.5$Å. Note that the data is taken at any instant along the trajectory when the desorbing molecule is located within the plane $Z_{\mathrm{CO}} \pm 0.5$Å. The plot at 2.5 Å is a clear demonstration of the high diffusivity over the surface plane. As remarked in this plot, the adsorbates that were initially at the adsorption sites (blue dots) distribute all over the surface during the dynamics (red dots). The also dense plots at 5.5 and 6.5 Å show that many CO are indeed transiently trapped in this region prior to desorption.

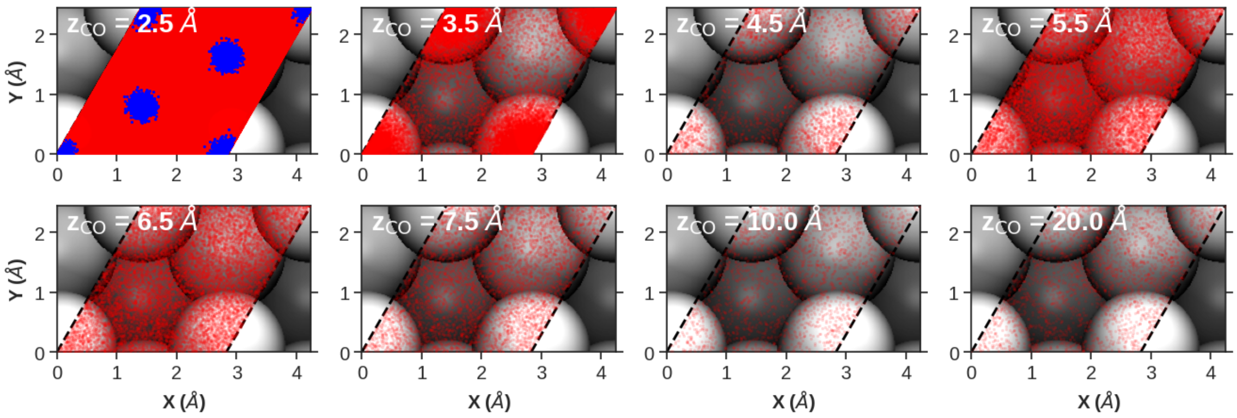

Figure S6: Center of mass coordinates over the surface taken whenever the desorbing CO locates at a height $Z_{\mathrm{CO}} \pm 0.5$Å (measured from the Pd topmost layer) at any instant along the whole trajectory. Results for 0.75 ML and an absorbed laser fluence of 80 J/m^2.

Figure S7 shows the polar angle distribution of the desorbing CO molecules for some of the high and low absorbed fluences that were calculated for each coverage. Together with the distributions calculated for all the desorbing molecules, we also show the distribution of the molecules that are identified as direct desorption (orange histograms) and transiently trapped desorption (green histograms). The rather broad distributions obtained for the molecules that follow 'direct desorption' remark the complexity of the desorption process that does not

follow the minimum energy paths in which the CO simply desorbs along the surface normal (see Figure S3). As expected, the transiently trapped molecules are characterized by broader polar-angle distributions.

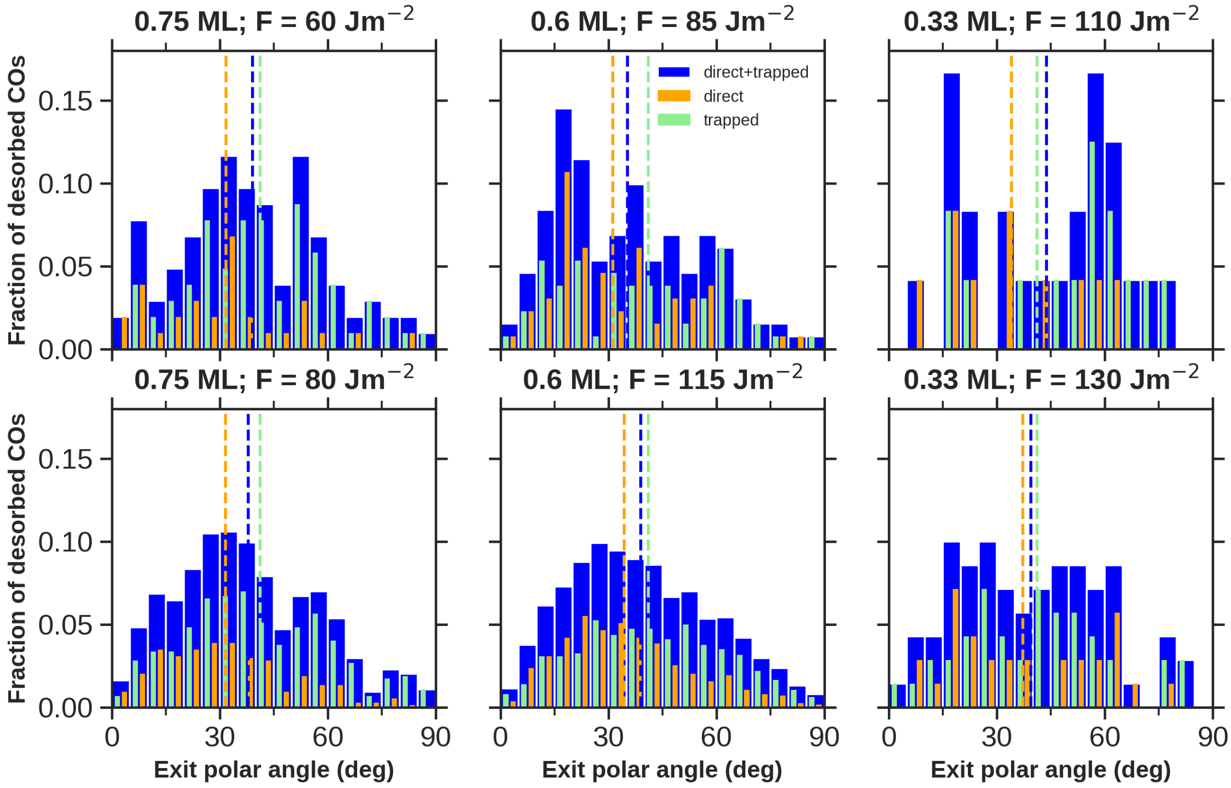

Figure S7: Polar angle distribution of the desorbing CO calculated for the coverage and laser fluence indicated on top of each plot (blue histograms). In each case, the angular distribution of the molecules that desorb directly (orange) and after being transiently trapped in the physisorption region (green) are shown separately. The mean polar angle for each distribution is plotted by a dashed line following the same color code.

Acknowledgement

The authors acknowledge financial support by the Gobierno Vasco-UPV/EHU [Project No. IT1569-22], by the Spanish MCIN/AEI/10.13039/501100011033 [Grant Nos. PID2019-107396GB-I00 and PID2022-140163NB-I00 together with FEDER "Una manera de hacer Europa"], and by the Education Department of the Basque Government [IKUR strategic

plan]. This research was conducted in the scope of the Transnational Common Laboratory (LTC) “QuantumChemPhys – Theoretical Chemistry and Physics at the Quantum Scale”. Computational resources were provided by the DIPC computing center.

References

(S1) Alducin, M.; Camillone, N.; Hong, S.-Y.; Juaristi, J. I. Electrons and Phonons Cooperate in the Laser-Induced Desorption of CO from Pd(111). *Phys. Rev. Lett.* **2019**, *123*, 246802.

(S2) Kresse, G.; Furthmüller, J. Efficiency of Ab-Initio Total Energy Calculations For Metals and Semiconductors Using a Plane-Wave Basis Set. *Comput. Mater. Sci.* **1996**, *6*, 15 – 50.

(S3) Kresse, G.; Furthmüller, J. Efficient Iterative Schemes For Ab Initio Total-Energy Calculations Using a Plane-Wave Basis Set. *Phys. Rev. B.* **1996**, *54*, 11169–11186.

(S4) Dion, M.; Rydberg, H.; Schröder, E.; Langreth, D. C.; Lundqvist, B. I. Van der Waals Density Functional for General Geometries. *Phys. Rev. Lett.* **2004**, *92*, 246401.

(S5) Blöchl, P. E. Projector Augmented-Wave Method. *Phys. Rev. B* **1994**, *50*, 17953.

(S6) Kresse, G.; Joubert, D. From Ultrasoft Pseudopotentials to the Projector Augmented-Wave Method. *Phys. Rev. B* **1999**, *59*, 1758–1775.

(S7) Methfessel, M.; Paxton, A. T. High-Precision Sampling for Brillouin-Zone Integration in Metals. *Phys. Rev. B* **1989**, *40*, 3616–3621.

(S8) Monkhorst, H. J.; Pack, J. D. Special Points for Brillouin-Zone Integrations. *Phys. Rev. B* **1976**, *13*, 5188–5192.

(S9) Hong, S.-Y.; Xu, P.; Camillone, N. R.; White, M. G.; Camillone, N. Adlayer Structure Dependent Ultrafast Desorption Dynamics in Carbon Monoxide Adsorbed on Pd (111). *J. Chem. Phys.* **2016**, *145*, 014704.

(S10) Tüshaus, M.; Berndt, W.; Conrad, H.; Bradshaw, A. M.; Persson, B. Understanding the Structure of High Coverage CO Adlayers. *Appl. Phys. A* **1990**, *51*, 91–98.

(S11) Zhang, Y.; Hu, C.; Jiang, B. Embedded Atom Neural Network Potentials: Efficient and Accurate Machine Learning with a Physically Inspired Representation. *J. Phys. Chem. Lett.* **2019**, *10*, 4962–4967.

(S12) Serrano-Jiménez, A.; Muzas, A. P. S.; Zhang, Y.; Ovčar, J.; Jiang, B.; Lončarić, I.; Juaristi, J. I.; Alducin, M. Photoinduced Desorption Dynamics of CO from Pd(111): A Neural Network Approach. *J. Chem. Theory Comput.* **2021**, *17*, 4648–4659.

(S13) Zhang, Y.-L.; Zhou, X.-Y.; Jiang, B. Accelerating the Construction of Neural Network Potential Energy Surfaces: A Fast Hybrid Training Algorithm. *Chin. J. Chem. Phys.* **2017**, *30*, 727–734.

(S14) Anisimov, S. I.; Kapeliovich B., L.; Perel'man, T. L. Electron Emission from Metal Surfaces Exposed to Ultrashort Laser Pulses. *Sov. Phys.-JETP* **1974**, *39*, 776–781.

(S15) Frischkorn, C.; Wolf, M. Femtochemistry at Metal Surfaces: Nonadiabatic Reaction Dynamics. *Chem. Rev.* **2006**, *106*, 4207–4233.

(S16) Saalfrank, P. Quantum Dynamical Approach to Ultrafast Molecular Desorption from Surfaces. *Chem. Rev.* **2006**, *106*, 4116–4159.

(S17) Szymanski, P.; Harris, A. L.; Camillone, N. Temperature-Dependent Electron-Mediated Coupling in Subpicosecond Photoinduced Desorption. *Surf. Sci.* **2007**, *601*, 3335–3349.

(S18) Shimizu, M.; Takahashi, T.; Katsuki, A. Magnetic Susceptibility and Electronic Specific Heat of Transition Metals and Alloys II. Pd Metal and Pd-Ag and Pd-Rh Alloys. *J. Phys. Soc. Jpn.* **1963**, *18*, 240–248.

(S19) Miiller, A. P.; Brockhouse, B. N. Anomalous Behavior of the Lattice Vibrations and the Electronic Specific Heat of Palladium. *Phys. Rev. Lett.* **1968**, *20*, 798–801.

(S20) Miiller, A. P.; Brockhouse, B. N. Crystal Dynamics and Electronic Specific Heats of Palladium and Copper. *Can. J. Phys.* **1971**, *49*, 704–723.

(S21) Veal, B. W.; Rayne, J. A. Heat Capacity of Palladium and Dilute Palladium: Iron Alloys from 1.4 to 100°K. *Phys. Rev.* **1964**, *135*, A442–A446.

(S22) Juaristi, J. I.; Alducin, M.; Díez Muiño, R.; Busnengo, H. F.; Salin, A. Role of Electron-Hole Pair Excitations in the Dissociative Adsorption of Diatomic Molecules on Metal Surfaces. *Phys. Rev. Lett.* **2008**, *100*, 116102.

(S23) Alducin, M.; Díez Muiño, R.; Juaristi, J. I. Non-Adiabatic Effects in Elementary Reaction Processes at Metal Surfaces. *Prog. Surf. Sci.* **2017**, *92*, 317 – 340.

(S24) Duffy, D. M.; Rutherford, A. M. Including the Effects of Electronic Stopping and Electron–ion Interactions in Radiation Damage Simulations. *J. Phys.: Condens. Matter* **2006**, *19*, 016207.
